# The ARIA® guidelines and the EUFOREA non-evidence-based algorithm cannot be compared and embedded in an integrated approach

**DOI:** 10.1007/s11882-025-01223-8

**Published:** 2025-10-10

**Authors:** Jean Bousquet, Bernardo Sousa-Pinto, G Walter Canonica, Ludger Klimek, Boleslaw Samolinski, Torsten Zuberbier

**Affiliations:** 1 Institute of Allergology, Charité, Universitätsmedizin Berlin, Corporate Member of Freie Universität Berlin and Humboldt-Universität zu Berlin, Berlin, Germany; 2 Fraunhofer Institute for Translational Medicine and Pharmacology ITMP, Immunology and Allergology, Berlin, Germany; 3 MASK-air, Montpellier, France; 4 MEDCIDS - Department of Community Medicine, Information and Health Decision Sciences, Faculty of Medicine, University of Porto, Porto, Portugal; 5 CINTESIS@RISE– Health Research Network, Faculty of Medicine, University of Porto, Porto, Portugal; 6 Department of Biomedical Sciences, Humanitas University, Pieve Emanuele, Milan, Italy; 7 Personalized Medicine, Asthma and Allergy, IRCCS Humanitas Research Hospital, Rozzano, Milan Italy; 8 Department of Otolaryngology, Head and Neck Surgery, Universitätsmedizin Mainz, Mainz, Germany; 9 Center for Rhinology and Allergology, Wiesbaden, Germany; 10 Department of Prevention of Environmental Hazards, Allergology and Immunology, Medical University of Warsaw, Warsaw, Poland

We read with interest a recent paper published in the journal [1]. However, we have major reservations that are discussed below.

Jean Bousquet started Allergic Rhinitis and its Impact on Asthma [ARIA®] in 1999 during a WHO workshop [2]. He is still leading it after developing five iterations of the guidelines. For the last iteration, ARIA® 2024-2025, ARIA® and the European Academy of Allergy and Clinical Immunology [EAACI] have started a collaboration. ARIA® has always been very successful, having involved more than 600 global experts in different fields from 92 countries [including not only allergists but also patient’s organisations, primary care physicians, pharmacists and policy makers, among others]. The ARIA® guidelines have always been developed with the best evidence-based models available at each time, and the 2017 iteration of ARIA® [3] was the case scenario of a JAMA review on the interpretation of guidelines [4]. Since 2017, there have been two new iterations of ARIA®: [i] ARIA® 2020, including for the first time evidence from patients’ behaviours collected using digital health tools [5], and [ii] ARIA® 2024-2025, as a person-centred, digitally enabled, artificial intelligence-assisted guideline [6]. We think that ARIA® is a true innovative evidence-based guideline from the first to the last iteration.

A non-evidence-based algorithm according to the Guidelines International Network [7] was published in 2021 by EUFOREA [8] and cannot be classified as a guideline. For example, Figure 1 – corresponding to the EUFOREA algorithm – has not been developed using an evidence-based method. Jean Bousquet was a member of the Executive Committee of EUFOREA, and one of the serious reasons for his departure was the improper methodology of these “recommendations”.

Therefore, the current paper[1] proposed the “integration” of a true guideline and an algorithm, neither of which according in many aspects [and sometimes disagreeing]. Furthermore, the proposal was done by consensus without indicating the methodology.

In addition to the methods underlying their development, there are other reasons why it is impossible to compare the ARIA® guidelines and the EUFOREA algorithm. ARIA® is always making new iterations that are currently being published [the latest one being ARIA® 2024-2025]. ARIA® 2024-2025 is, for the first time in chronic diseases, a true person-centred [and not patient-centred], digitally enabled, artificial intelligence-assisted guideline [6]. Its recommendations will not only be supported by a set of systematic reviews [9–14] but also by findings from studies using MASK-air® [for review of original papers [15]], one of the two apps referenced by the Organisation of Economic Cooperation and Development [OECD] [16]. The novel ARIA® 2024-2025 guideline will bring major clinical and methodological innovations, which will include [i] incorporating values and preferences [17, 18], costs [19], digital health, and planetary health [20–22] for the development of recommendations, [ii] considering different sources of data, including mHealth and pharmacovigilance data, [iii] having a person-centred approach. The latter will be achieved through [i] the use of digital health [MASK-air® data], web searches and AI [23] for question generation, and [ii] considering adherence [24], onset of action and satisfaction to treatments [25]. Over 15 papers providing evidence supporting the underlying recommendations have already been published and many more will be published over the next few months. The paper proposing a comparison between ARIA® and EUFOREA is not taking into account these recent developments.

Finally, the EUFOREA statements do not necessarily report on the conflicts of interest [CoI] of authors. In fact, CoI have not been listed in this paper [1] nor in the EUFOREA algorithm that is apparently the basis for this paper [8]. The Guidelines International Network [GIN] has issued a set of principles for disclosure of interests and management of conflicts in guidelines [26]. The authors of these principles recognise that “CoI create a risk of bias in decisions or recommendations [so that] systematic approaches to the disclosure of interests and CoI management are necessary to minimise potential bias”. By not reporting the authors’ CoI, EUFOREA is not only not complying with the GIN principle #4 [“A guideline development group should disclose interests publicly, including all direct financial and indirect CoI, and these should be easily accessible for users of the guideline.”] but also precluding a transparent assessment of the other principles. The current paper was sponsored by a single drug company from Malaysia and the need for funding has not been sufficiently disclosed.

In conclusion, we think that this paper is misleading since there cannot be an integrated approach between the ARIA® guidelines and the EUFOREA algorithm, particularly as the latter is non-evidence-based and has already been outdated by ARIA 2020 and the novel ARIA 2024-2025.

## Data Availability

No datasets were generated or analysed during the current study.
